# *N*,*N*′-(Ethane-1,2-di­yl)bis­(methane­sulfon­amide)

**DOI:** 10.1107/S1600536814000622

**Published:** 2014-01-15

**Authors:** Wesley Ting Kwok Chan, Ka Yan Karen Kung, Man-kin Wong

**Affiliations:** a Laboratory of X-Ray Crystal Structure Analysis, Department of Applied Biology and Chemical Technology, The Hong Kong Polytechnic University, Hung Hom, Hong Kong S.A.R.; b State Key Laboratory of Chirosciences and Department of Applied Biology and Chemical Technology, The Hong Kong Polytechnic University, Hung Hom, Hong Kong S.A.R.

## Abstract

The mol­ecular structure of the title compound, C_4_H_12_N_2_O_4_S_2_, has crystallographic inversion symmetry. The central N—C—C—N moiety was refined as disordered over two sets of sites with an approximate occupancy ratio of 3:1 [0.742 (15):0.258 (15). In the crystal, N—H⋯O hydrogen bonds link adjacent mol­ecules into a thick sheet structure parallel to the *b*-axis direction.

## Related literature   

For analogous disulfonamide compounds, see: Al-Dajani *et al.* (2011*a* ▶,*b* ▶). For other analyses and properties of disulfonamide compounds, see: Alyar *et al.* (2011 ▶, 2012 ▶). For their biological and pharmaceutical activity, see: Sahu *et al.* (2007 ▶); Innocenti *et al.* (2008 ▶). For a description of the Cambridge Structural Database, see: Allen (2002 ▶).
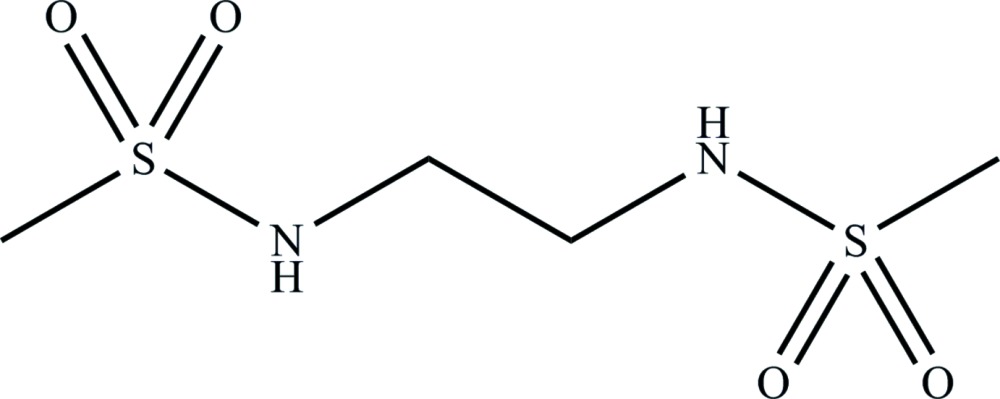


## Experimental   

### 

#### Crystal data  

C_4_H_12_N_2_O_4_S_2_
*M* *_r_* = 216.28
Monoclinic, 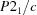

*a* = 10.5668 (11) Å
*b* = 5.6092 (6) Å
*c* = 8.5141 (9) Å
β = 109.790 (6)°
*V* = 474.84 (9) Å^3^
*Z* = 2
Mo *K*α radiation
μ = 0.54 mm^−1^
*T* = 296 K
0.30 × 0.28 × 0.10 mm

#### Data collection  

Bruker APEXII CCD area-detector diffractometer
Absorption correction: multi-scan (*SADABS*; Bruker, 2001 ▶) *T*_min_ = 0.854, *T*_max_ = 0.948
10251 measured reflections
1115 independent reflections
1033 reflections with *I* > 2σ(*I*)
*R*_int_ = 0.025

#### Refinement  

*R*[*F* ^2^ > 2σ(*F* ^2^)] = 0.032
*wR*(*F* ^2^) = 0.099
*S* = 1.10
1115 reflections
76 parameters
2 restraints
H-atom parameters constrained
Δρ_max_ = 0.31 e Å^−3^
Δρ_min_ = −0.32 e Å^−3^

### 

Data collection: *APEX2* (Bruker, 2007 ▶); cell refinement: *SAINT* (Bruker, 2007 ▶); data reduction: *SAINT*; program(s) used to solve structure: *SHELXTL* (Sheldrick, 2008 ▶); program(s) used to refine structure: *SHELXTL*; molecular graphics: *SHELXTL*; software used to prepare material for publication: *SHELXTL*.

## Supplementary Material

Crystal structure: contains datablock(s) I. DOI: 10.1107/S1600536814000622/nk2217sup1.cif

Structure factors: contains datablock(s) I. DOI: 10.1107/S1600536814000622/nk2217Isup2.hkl

Supporting information file. DOI: 10.1107/S1600536814000622/nk2217Isup3.mol

Supporting information file. DOI: 10.1107/S1600536814000622/nk2217Isup4.cdx

Supporting information file. DOI: 10.1107/S1600536814000622/nk2217Isup5.cml

CCDC reference:

Additional supporting information:  crystallographic information; 3D view; checkCIF report

## Figures and Tables

**Table 1:** Hydrogen-bond geometry (Å, °)

*D*—H⋯*A*	*D*—H	H⋯*A*	*D*⋯*A*	*D*—H⋯*A*
N1—H1⋯O1^i^	0.86	2.46	3.009 (6)	122

Symmetry code: (i) 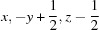.

**Fig. 1.**
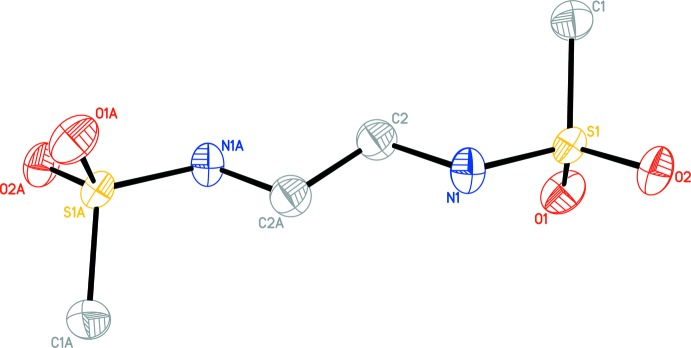
Molecular structure of the title compound, with the ellipsoids drawn at 30% probability. Hydrogen atoms and disordered components are omitted in the figure. (Symmetry equilvalent atoms generated by -x, -y, -z)

**Fig. 2.**
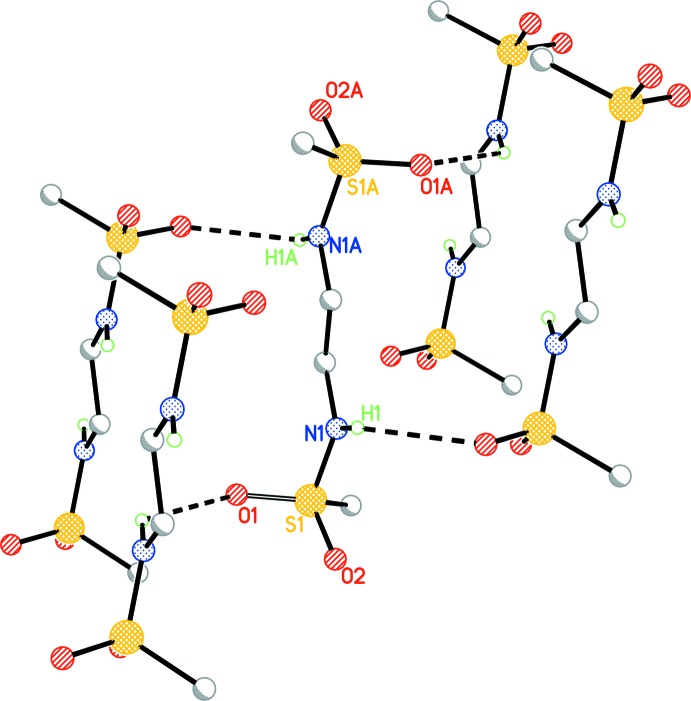
Hydrogen bonding observed in the lattice, and only hydrogen bonding protons are shown. Disorder components are omitted. (Symmetry equivalent atoms generated by -x, -y, -z, -x, 1/2 + y, 0.5 - z and +x, -0.5 - y, 1/2 + z)

**Fig. 3.**
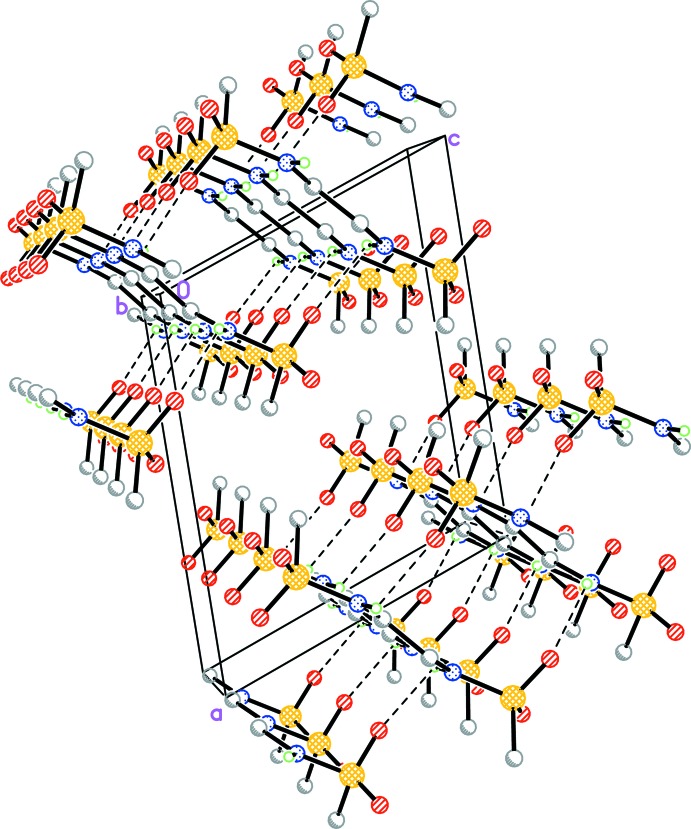
Packing diagram (viewed slightly off b-axis) showing hydrogen bonds (dashed lines). Disordered components are omitted.

## supplementary crystallographic information

### 1. Comment

Sulfonamides have long been utilised as bacteriostatic agents in both human and veterinary medicine, and their derivatives have a wide range of pharmacological applications (Alyar *et el.*, 2011, 2012). In recent years, concentrated effort has been made on the effectiveness of the disulfonamide compounds as antimicrobial agents, and reported here is the solid state structure of a related disulfonamide intended for further studies: N,N'-ethane-1,2-diyl-bis(methane-sulfonamide) (Figure 1). The asymmetric unit of the title consists of half of the molecule, as the central C-C bond sits on top of an inversion centre. This structure is directly analogous to several previously reported structures (e.g. Al-Dajani *et al.*, 2011*a*,*b*; entries AYONOS and AYORUC in the Cambridge Structural Database (version 5.34, Allen (2002))), which all contains aryl sulfonamide moieties. Unlike these reported structures, the methyl group does not exert significant steric pressure on the neighbouring sulfur atom. While the bond angle surrounding the sulfonamide S atom ranges between 106.80 (11)° and 117.60 (13)°, the overall geometry does not significantly deviate from an ideal tetrahedral configuration (average bond angle = 109.4°). The molecule is also slightly twisted at the N atom (C1-S1-N1-C2 torsion angle = -56.19°). An extended network is formed through intramolecular hydrogen bonding. Any one molecule is hydrogen-bonded to four different neighbouring molecules (Figure 2, Table 1).

### 2. Experimental

THF solution of 1,2-diaminoethane was added dropwise to the tetrahydrofuran (THF) solution of methyl sufonyl chloride in equimolar fashion, with the temperature maintained between -5 and -10°C. The reaction mixture was allowed to warm and stirred for 24 h at room temperature. Upon completion of the reaction, the solvent was removed under vacuum, and the solid residue was purified by column chromatography. The product was identified with ^1^H-NMR. To recrystallize, the product was disolved in minimum amount of hot dichloromethane (DCM), and colourless, plate-like crystals formed upon gradual cooling of the solution.

Please note that a polymorph (deposited with the Cambridge Crystallographic Data centre as CCDC 958590) of the title compound was obtained initially. Crystals of this polymorph were obtained at elevated temperature, in a complexation reaction using the title compound as a ligand. Unfortunately, the crystallization condition cannot be accurately identified, and independent crystallization using the condition stated gave the structure reported in this manuscript.

### 3. Refinement

Minor rotational disorder was modelled and the refined occupancy ratio is 0.742:0.258 (15). The SHELXL constraint RIGU was used for the disordered carbon of the minor component, and SADI to restrain the refinements of S—N' and N'-C' bond lengths. All protons were refined using suitable riding models. Terminal C—H bonds, ethyl C—H bonds and amido N—H bonds were assumed to be 0.960 Å, 0.970 Å and 0.860 Å, respectively. The *U* values of terminal methyl protons are set to be 1.5 times of that of the attached carbon, while all other protons are calculated to be 1.2 times of the *U* values of the attached atom.

### Crystal data

**Table:**

C_4_H_12_N_2_O_4_S_2_	*F*(000) = 228
*M**_r_* = 216.28	*D*_x_ = 1.513 Mg m^−^^3^
Monoclinic, *P*2_1_/*c*	Mo *K*α radiation, λ = 0.71073 Å
*a* = 10.5668 (11) Å	Cell parameters from 5915 reflections
*b* = 5.6092 (6) Å	θ = 2.7–27.7°
*c* = 8.5141 (9) Å	µ = 0.54 mm^−^^1^
β = 109.790 (6)°	*T* = 296 K
*V* = 474.84 (9) Å^3^	Plate, colourless
*Z* = 2	0.30 × 0.28 × 0.10 mm

### Data collection

**Table:**

Bruker APEXII CCD area-detector diffractometer	1033 reflections with *I* > 2σ(*I*)
phi and ω scans	*R*_int_ = 0.025
Absorption correction: multi-scan (*SADABS*; Bruker, 2001)	θ_max_ = 27.7°, θ_min_ = 2.1°
*T*_min_ = 0.854, *T*_max_ = 0.948	*h* = −13→13
10251 measured reflections	*k* = −7→7
1115 independent reflections	*l* = −11→11

### Refinement

**Table:**

Refinement on *F*^2^	Hydrogen site location: inferred from neighbouring sites
Least-squares matrix: full	H-atom parameters constrained
*R*[*F*^2^ > 2σ(*F*^2^)] = 0.032	*w* = 1/[σ^2^(*F*_o_^2^) + (0.0579*P*)^2^ + 0.1493*P*] where *P* = (*F*_o_^2^ + 2*F*_c_^2^)/3
*wR*(*F*^2^) = 0.099	(Δ/σ)_max_ < 0.001
*S* = 1.10	Δρ_max_ = 0.31 e Å^−^^3^
1115 reflections	Δρ_min_ = −0.32 e Å^−^^3^
76 parameters	Extinction correction: *SHELXTL* (Sheldrick, 2008), Fc^*^=kFc[1+0.001xFc^2^λ^3^/sin(2θ)]^-1/4^
2 restraints	Extinction coefficient: 0.060 (8)

### Special details

**Table:**

Geometry. All e.s.d.'s (except the e.s.d. in the dihedral angle between two l.s. planes) are estimated using the full covariance matrix. The cell e.s.d.'s are taken into account individually in the estimation of e.s.d.'s in distances, angles and torsion angles; correlations between e.s.d.'s in cell parameters are only used when they are defined by crystal symmetry. An approximate (isotropic) treatment of cell e.s.d.'s is used for estimating e.s.d.'s involving l.s. planes.
Refinement. Refinement of *F*^2^ against ALL reflections. The weighted *R*-factor *wR* and goodness of fit *S* are based on *F*^2^, conventional *R*-factors *R* are based on *F*, with *F* set to zero for negative *F*^2^. The threshold expression of *F*^2^ > σ(*F*^2^) is used only for calculating *R*-factors(gt) *etc*. and is not relevant to the choice of reflections for refinement. *R*-factors based on *F*^2^ are statistically about twice as large as those based on *F*, and *R*- factors based on ALL data will be even larger.A disorder of the N—C—C—N chain in the molecule is observed. The occupancies were allowed to be refined freely. SADI restrains were used on the bond lengths of the *S*-N bond and the N—C bond. Furthermore, the hard constrain RIGU was placed on the displacement parameters of C2'. ISOR did not significantly improve the refinement.

### Fractional atomic coordinates and isotropic or equivalent isotropic displacement parameters (Å^2^)

**Table:**

	*x*	*y*	*z*	*U*_iso_*/*U*_eq_	Occ. (<1)
S1	0.27263 (4)	0.10237 (7)	0.80849 (5)	0.0399 (2)	
O1	0.22112 (17)	−0.0101 (3)	0.92544 (17)	0.0616 (4)	
O2	0.34539 (14)	0.3196 (2)	0.85836 (18)	0.0564 (4)	
C1	0.3769 (2)	−0.1041 (4)	0.7557 (3)	0.0599 (5)	
H1A	0.4547	−0.1357	0.8519	0.090*	
H1B	0.4046	−0.0397	0.6681	0.090*	
H1C	0.3282	−0.2497	0.7184	0.090*	
N1	0.1439 (6)	0.1585 (10)	0.6441 (5)	0.0419 (11)	0.742 (15)
H1	0.1218	0.3021	0.6106	0.050*	0.742 (15)
C2	0.0667 (4)	−0.0467 (5)	0.5549 (6)	0.0531 (12)	0.742 (15)
H2A	0.0546	−0.1622	0.6334	0.064*	0.742 (15)
H2B	0.1134	−0.1235	0.4882	0.064*	0.742 (15)
N1'	0.162 (2)	0.170 (3)	0.637 (2)	0.063 (5)	0.258 (15)
H1'	0.1771	0.2772	0.5731	0.076*	0.258 (15)
C2'	0.0165 (18)	0.026 (4)	0.5855 (12)	0.069 (5)	0.258 (15)
H2'A	0.0255	−0.1192	0.6502	0.082*	0.258 (15)
H2'B	−0.0520	0.1255	0.6046	0.082*	0.258 (15)

### Atomic displacement parameters (Å^2^)

**Table:**

	*U*^11^	*U*^22^	*U*^33^	*U*^12^	*U*^13^	*U*^23^
S1	0.0482 (3)	0.0379 (3)	0.0328 (3)	−0.00469 (15)	0.01254 (19)	−0.00383 (13)
O1	0.0881 (10)	0.0592 (9)	0.0456 (8)	−0.0079 (8)	0.0331 (7)	0.0037 (6)
O2	0.0614 (8)	0.0464 (8)	0.0533 (8)	−0.0136 (6)	0.0088 (6)	−0.0113 (6)
C1	0.0590 (12)	0.0546 (12)	0.0642 (13)	0.0071 (9)	0.0185 (10)	−0.0093 (9)
N1	0.0397 (18)	0.0455 (19)	0.0334 (14)	−0.0088 (13)	0.0030 (11)	0.0088 (13)
C2	0.0401 (18)	0.0432 (13)	0.063 (2)	0.0043 (11)	0.0005 (15)	−0.0143 (12)
N1'	0.043 (6)	0.051 (7)	0.090 (11)	−0.003 (5)	0.015 (5)	−0.035 (7)
C2'	0.064 (7)	0.092 (11)	0.051 (5)	−0.034 (8)	0.021 (5)	−0.006 (5)

### Geometric parameters (Å, º)

**Table:**

S1—O2	1.4267 (13)	N1—H1	0.8600
S1—O1	1.4327 (14)	C2—C2^i^	1.498 (6)
S1—N1'	1.574 (13)	C2—H2A	0.9700
S1—N1	1.619 (4)	C2—H2B	0.9700
S1—C1	1.758 (2)	N1'—C2'	1.66 (3)
C1—H1A	0.9600	N1'—H1'	0.8600
C1—H1B	0.9600	C2'—C2'^i^	1.41 (2)
C1—H1C	0.9600	C2'—H2'A	0.9700
N1—C2	1.465 (6)	C2'—H2'B	0.9700
			
O2—S1—O1	117.55 (9)	S1—N1—H1	121.5
O2—S1—N1'	103.0 (7)	N1—C2—C2^i^	106.8 (3)
O1—S1—N1'	114.5 (10)	N1—C2—H2A	110.4
O2—S1—N1	107.6 (2)	C2^i^—C2—H2A	110.4
O1—S1—N1	106.3 (2)	N1—C2—H2B	110.4
O2—S1—C1	108.52 (10)	C2^i^—C2—H2B	110.4
O1—S1—C1	107.80 (11)	H2A—C2—H2B	108.6
N1'—S1—C1	104.7 (7)	S1—N1'—C2'	117.2 (14)
N1—S1—C1	108.8 (2)	S1—N1'—H1'	121.4
S1—C1—H1A	109.5	C2'—N1'—H1'	121.4
S1—C1—H1B	109.5	C2'^i^—C2'—N1'	104.9 (14)
H1A—C1—H1B	109.5	C2'^i^—C2'—H2'A	110.8
S1—C1—H1C	109.5	N1'—C2'—H2'A	110.8
H1A—C1—H1C	109.5	C2'^i^—C2'—H2'B	110.8
H1B—C1—H1C	109.5	N1'—C2'—H2'B	110.8
C2—N1—S1	116.9 (3)	H2'A—C2'—H2'B	108.8
C2—N1—H1	121.5		
			
O2—S1—N1—C2	170.3 (5)	O2—S1—N1'—C2'	−149.8 (15)
O1—S1—N1—C2	−63.0 (6)	O1—S1—N1'—C2'	−21.0 (18)
N1'—S1—N1—C2	113 (6)	N1—S1—N1'—C2'	−25 (5)
C1—S1—N1—C2	52.9 (6)	C1—S1—N1'—C2'	96.8 (17)
S1—N1—C2—C2^i^	162.7 (5)	S1—N1'—C2'—C2'^i^	−138 (2)

Symmetry code: (i) −*x*, −*y*, −*z*+1.

### Hydrogen-bond geometry (Å, º)

**Table:**

*D*—H···*A*	*D*—H	H···*A*	*D*···*A*	*D*—H···*A*
N1—H1···O1^ii^	0.86	2.46	3.009 (6)	122

Symmetry code: (ii) *x*, −*y*+1/2, *z*−1/2.
